# A comparison of anti‐arrhythmic efficacy of carvedilol vs metoprolol succinate in patients with implantable cardioverter‐defibrillators

**DOI:** 10.1002/clc.23144

**Published:** 2019-01-14

**Authors:** Mohamed Ayan, Fuad Habash, Bilal Alqam, Zaid Gheith, Michael Cross, Srikanth Vallurupalli, Hakan Paydak

**Affiliations:** ^1^ Division of Cardiology University of Arkansas for Medical Sciences Little Rock Arkansas

**Keywords:** appropriate therapy, cardiomyopathy, carvedilol, implantable cardioverter‐defibrillator, inappropriate therapy, metoprolol succinate

## Abstract

**BACKGROUND:**

The effects of carvedilol and metoprolol succinate on appropriate and inappropriate implantable cardioverter defibrillator (ICD) therapy in patients with heart failure with reduced ejection fraction (HFrEF) are not fully understood.

**HYPOTHESIS:**

The hypothesis of our study is possible carvedilol superiority over metoprolol in patients with ICD.

**METHODS:**

All patients with ICD registered to a single device clinic between 1/2012 and 6/2017 (n = 569) were identified. Patients with systolic heart failure (left ventricular ejection fraction ≤40%) treated with carvedilol vs metoprolol succinate were compared. Primary endpoint was difference in survival free of appropriate device therapy (shock or anti‐tachycardia pacing, ATP). Secondary endpoints were freedom from inappropriate therapy (shock or ATP) and all cause death.

**RESULTS:**

A total of 225 patients were included in the analysis with median follow up of 57 months (IQR 33.7‐90). The 2 groups were comparable in the baseline characteristics. Carvedilol was superior to metoprolol succinate in improving survival free of appropriate ICD therapy (HR 0.42; 95% CI 0.24‐0.72, *P* = 0.01). This difference was driven by reduction in survival free of appropriate shocks (HR 0.30; 95% CI 0.15‐0.63, *P* = −0.01) while there was no significant difference in appropriate ATP (HR 0.55; 95% CI 0.28‐1.1, *P* = 0.12). There was no significant difference in time to inappropriate shocks (HR 1.02; 95% CI 0.19‐5.6, *P* = 0.97), inappropriate ATP (HR 0.93, OR 0.24‐3.5, p value 0.9) or all cause death (HR 0.8; 95% CI 0.42‐1.5, *P* = 0.52).

**CONCLUSIONS:**

This study suggests that carvedilol use was associated with improved survival free of appropriate ICD therapy compared to metoprolol succinate in patients with HFrEF.

**Abbreviations**
ATP: anti‐tachycardia pacing
CM: cardiomyopathy
CRT: cardiac resynchronization therapy
HF: heart failure
HFrEF: heart failure with reduced ejection fraction
HR: hazard ratio
ICD: implantable cardioverter‐defibrillator
LVEF: left ventricular ejection fraction
VT: ventricular tachycardia

## 1 INTRODUCTION

The implantable cardioverter‐defibrillator (ICD) is an important tool in the prevention of sudden cardiac death due to arrhythmias in patients with heart failure (HF) with heart failure with reduced ejection fraction (HFrEF). However, appropriate and inappropriate ICD therapies result in pain and subsequent psychological apprehension, anxiety, and impaired quality of life.^1^, ^2^, ^3^ Furthermore recurrent inappropriate shocks may lead to worsening HF.^4^, ^5^ Medical therapy for HFrEF includes beta adrenergic blockers of which three (carvedilol, metoprolol succinate, and bisoprolol) have been shown to improve mortality.^6^, ^7^, ^8^ There have been no direct comparative trials to support the use of one beta‐blocker over the other in HFrEF, specifically in reducing cardiac arrhythmias and thus preventing ICD therapies. The aim of the current study was to compare the antiarrhythmic efficacy of carvedilol and metoprolol succinate in the treatment in HFrEF in patients with an ICD.

## 2 METHODS

### 2.1 Study design

**Figure 1:**
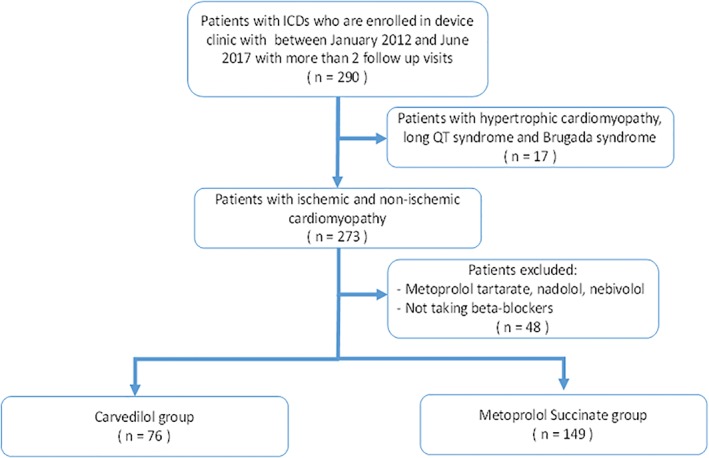
Process of subject identification and inclusion in the study are depicted in the flowchart

All patients with an ICD (including cardiac resynchronization therapy [CRT]) who were followed at the device clinic at University of Arkansas between January 2012 and June 2017 were screened in this retrospective study (*n* = 569). Exclusion criteria included (a) patients who had less than two follow‐up visits were excluded (*n* = 279), (b) patients with hypertrophic cardiomyopathy (CM), Brugada syndrome, and long QT syndrome (*n* = 17), and (c) patients who were on beta‐blockers that are not proven to improve outcomes in HFrEF (*n* = 48). (Figure 1) Patients were assigned either to carvedilol or metoprolol succinate group based on the beta blocker at the time of device implantation. Baseline demographic and clinical data were collected at the time of device implant from review of medical record. The primary endpoint was survival free of appropriate device therapy (shock or anti‐tachycardia pacing [ATP]). Appropriate shock or ATP was defined as therapies administered to treat ventricular arrhythmias. Secondary endpoints included survival free of inappropriate therapy (shock or ATP) and all cause death. Inappropriate ICD therapies were defined as those administered by the device to treat any supraventricular arrhythmias. Events were adjudicated based on record review and correlated with intracardiac electrogram tracings when available. To control for differences in dosage, groups were further stratified based on widely accepted dose equivalency conversion (25 mg carvedilol = 100 mg metoprolol succinate).

Statistical analysis was performed using MedCalc (version 18, Ostend, Belgium). Categorical variables were depicted using percentages and compared using *χ* ^2^ test for while continuous variables were described by mean +/− SD and compared using *t* tests. Kaplan‐Meier analysis was performed to compare survival free of primary and secondary endpoints, and the survival curves were compared using log rank test. To control for other risk factors for arrhythmias in this population (chosen based on widely accepted risk and significance on univariate analysis *P* < 0.1), Cox regression analysis was performed. A *P*‐value <0.05 was considered statistically significant.

## 3 RESULTS

A total of 225 patients were included in the analysis (Figure 1). Table 1 depicts baseline characteristics of the cohort. There was no significant difference in age at implantation, gender distribution, race, or baseline comorbidities. The left ventricular ejection fraction (LVEF) was similar at time of implantation and there was no significant difference in ischemic CM or history of ventricular arrhythmias (secondary prevention ICD). Mean dose of metoprolol succinate was 126 ± 86 mg/day(median 100 mg, IQR 50‐200) and for carvedilol was 37 ± 19 mg /day (median 50 mg, IQR 25‐50). When compared based on dose equivalency, significantly more patients in carvedilol group received higher doses (50 carvedilol vs 200 mg metoprolol, 57.8% vs 37.8%, *P* = 0.04; 25 carvedilol vs 100 mg metoprolol 18.4% vs 20.1%, p = NS; 12.5 carvedilol vs 50 mg metoprolol 17.1% vs 19.3%, p = NS; 6.25 carvedilol vs 25 mg metoprolol 6.6% vs 15.9%, *P* = 0.0007). CRT accounted for one third of the devices. Less than 10% of the cohorts were on anti‐arrhythmic agents at the time of implantation and use was equally distributed between the two groups.

**Table 1:** Baseline characteristics of the cohort

		Carvedilol (*n* = 76)	Metoprolol succinate (*n* = 149)	*P* value
Male gender		48 (63.2)	87 (58.4)	0.490
Race				0.174
	White	35 (46.1)	82 (55.0)	
	African American	40 (52.6)	63 (42.3)	
	Hispanic	0 (0.0)	3 (2.0)	
BMI		30.7 ± 6.9	30.6 ± 8.0	0.972
Age at implantation		58.1 ± 13.8	58.9 ± 12.4	0.634
Type of ICD				0.740
	Single chamber	31 (40.8)	69 (46.3)	
	Dual chamber	20 (26.3)	34 (22.8)	
	CRT	24 (31.6)	46 (30.9)	
Indication for ICD				0.709
	Primary	69 (90.8)	132 (88.6)	
	Secondary	7 (9.2)	16 (10.7)	
Ejection fraction at implantation		25.1 ± 7.2	24.8 ± 9.0	0.827
MI		23 (30.3)	49 (32.9)	0.690
CABG		14 (18.4)	29 (19.5)	0.851
Diabetes mellitus		29 (38.2)	53 (35.6)	0.703
Atrial arrhythmias		19 (25%)	55 (37%)	0.07
Cardiomyopathy				0.965
	Ischemic	37 (48.7)	73 (49.0)	
	Nonischemic	39 (51.3)	76 (51.0)	
Digoxin		19 (25.0)	40 (26.8)	0.766
ACE/ARB		61 (80.3)	103 (69.1)	0.076
Aldosterone antagonist		34 (44.7)	64 (43.0)	0.799
Diuretic		60 (78.9)	116 (77.9)	0.851
Statin		50 (65.8)	97 (65.1)	0.918
Anti‐arrhythmic agents at device implant		7 (9.2%)	12 (8.1%)	0.8

Abbreviations: ACE, angiotensin converting enzyme inhibitor; ARB, angiotensin receptor blocker; BMI, body mass index; CABG, coronary artery bypass graft; ICD, implantable cardioverter‐defibrillator; MI, myocardial infarction.

**Figure 2:**
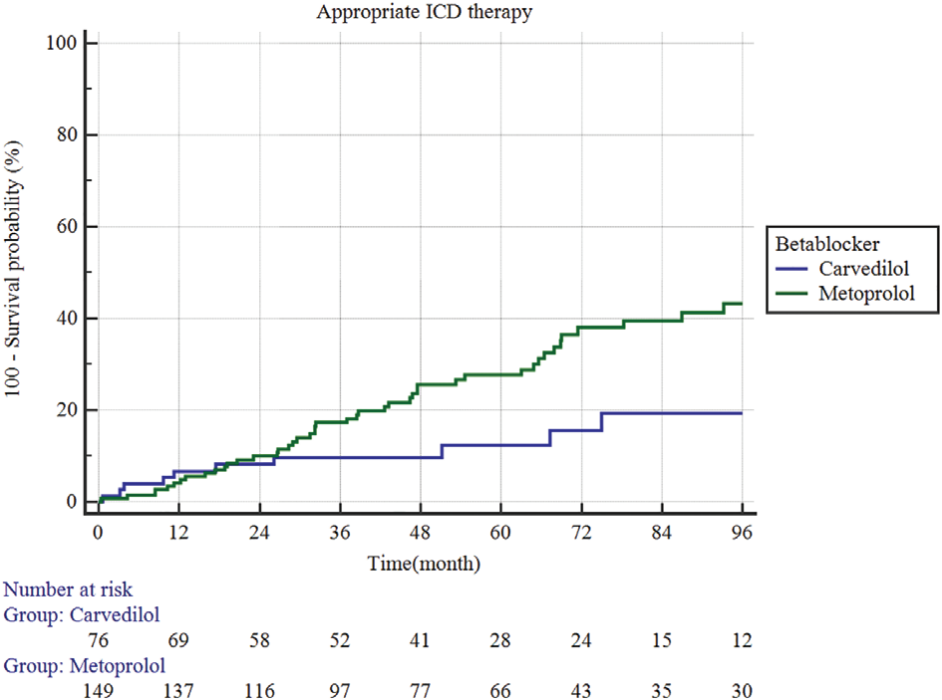
Carvedilol was superior to metoprolol succinate in promoting survival free of appropriate implantable cardioverter defibrillator (ICD) therapy (hazard ratio [HR] 0.42; 95% confidence interval [CI] 0.24‐0.72, *P* = 0.01)

**Figure 3:**
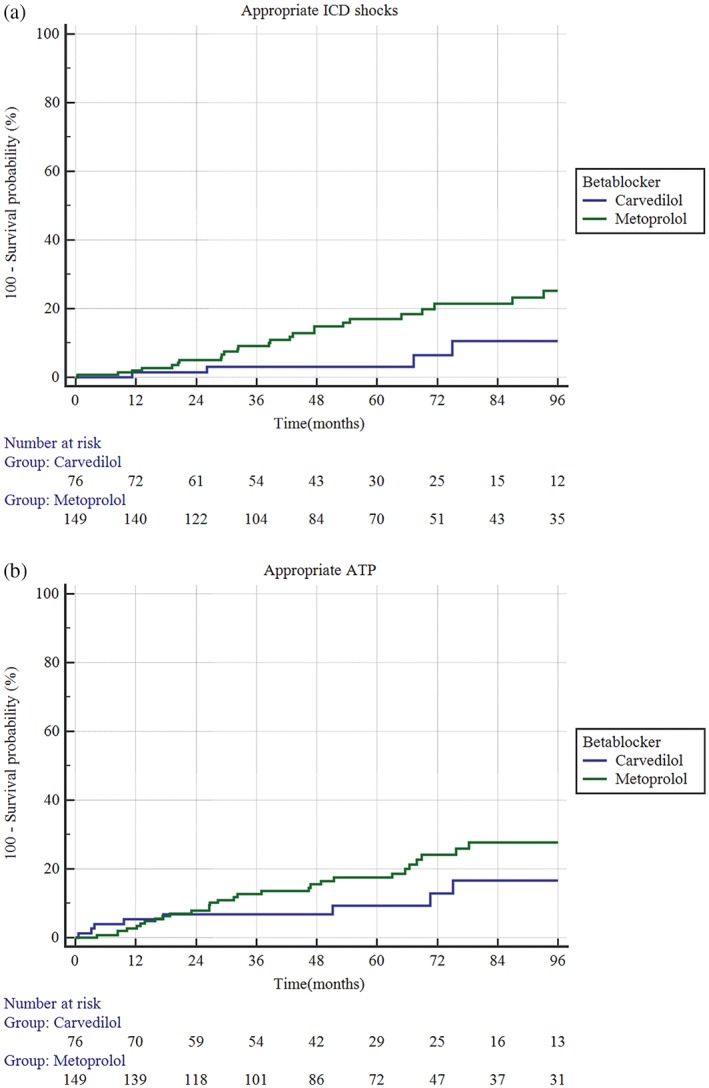
A, carvedilol was superior to metoprolol succinate in preventing appropriate implantable cardioverter defibrillator (ICD) shocks (hazard ratio [HR] 0.30; 95% confidence interval [CI] 0.15‐0.63, *P* = 0.01); B, there was no significant difference between carvedilol and metoprolol succinate in preventing appropriate anti‐tachycardia pacing (ATP) (HR 0.55; 95% CI 0.28‐1.1, *P* = 0.12)

Median follow‐up was 57 months (IQR 33.7‐90 months). Over this period, 27.2% (*n* = 72) of the cohort received appropriate ICD therapies and all‐cause mortality was 19.5% (*n* = 53). By Kaplan‐Meier analysis, carvedilol was superior to metoprolol succinate in improving survival free of appropriate ICD therapy (hazard ratio [HR] 0.42; 95% confidence interval [CI] 0.24‐0.72, *P* = 0.01) (Figure 2). This difference was driven by reduction in survival free of appropriate shocks (HR 0.30; 95% CI 0.15‐0.63, *P* = 0.01) (Figure 3A) while there was no significant difference in appropriate ATP (HR 0.55; 95% CI 0.28‐1.1, *P* = 0.12) (Figure 3B). There was no significant difference in survival free of inappropriate shocks (HR 1.02; 95% CI 0.19‐5.6, *P* = 0.97), inappropriate ATP (HR 0.93, OR 0.24‐3.5, *P* value 0.9) or all cause death (HR 0.8; 95% CI 0.42‐1.5, *P* = 0.52). In Cox regression analysis (including age, aldosterone antagonist use, digoxin use, anti‐arrhythmic drug use, type of CM, history of atrial arrhythmias, history of ventricular arrhythmias, type of beta‐blocker, LVEF at implantation and dose of beta blocker), carvedilol use (odds ratio [OR] 0.48, 95% CI 0.23‐0.96, *P* = 0.04) and an LVEF >25% (OR 0.42, p5% CI 0.24‐0.75, *P* = 0.008) predicted a lower risk for appropriate ICD therapies while baseline antiarrhythmic use predicted a higher risk (OR 2.8, 95% CI 1.6‐4.9, *P* = 0.0002).

**Figure 4:**
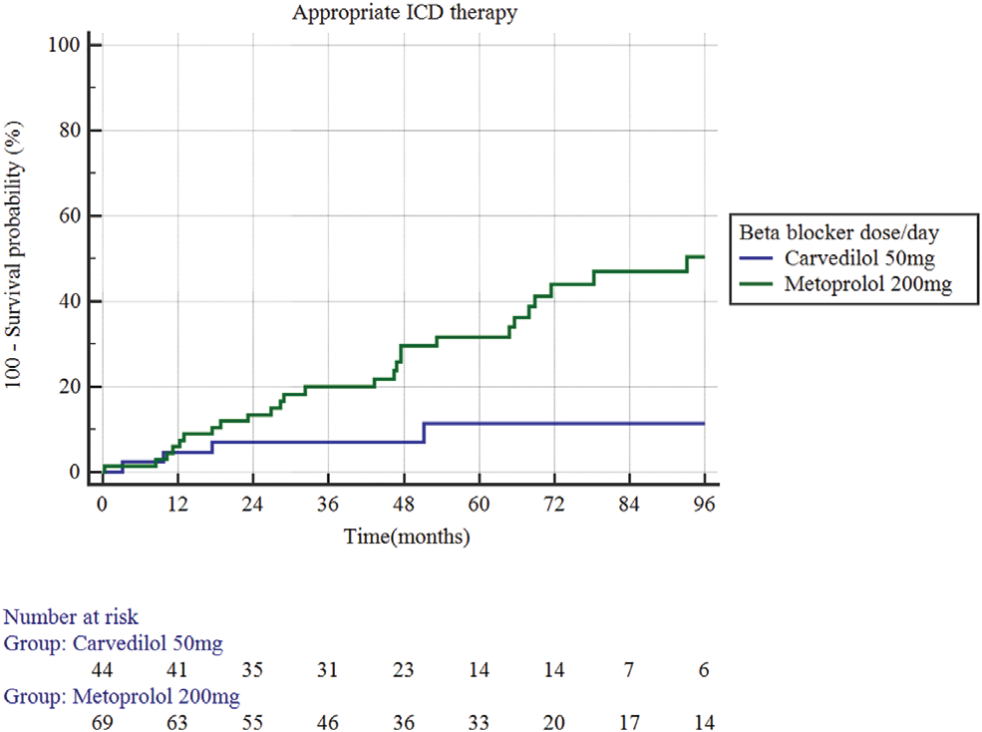
At equivalent doses, carvedilol remained superior to metoprolol succinate in promoting survival free of appropriate therapies (odds ratio [OR] 0.26, 95% confidence interval [CI] 0.12‐0.55, *P* = 0.007)

There was no significant difference in survival free of appropriate ICD therapies between the two drugs at carvedilol equivalent doses of 6.25, 12.5, and 25 mg daily though the number of patients in each subgroup was likely too small to detect a significant difference. At higher dose equivalent (50 mg carvedilol and 200 mg metoprolol), carvedilol was associated with a significantly lower risk of appropriate ICD therapies (OR 0.26, 95% CI 0.12‐0.55, *P* = 0.007, Figure 4).

## 4 DISCUSSION

The major finding of this study was that carvedilol was superior in preventing appropriate device therapies (mainly ICD shocks) compared to metoprolol succinate in patients with HFrEF even after adjusting for other risk factors including a history of ventricular arrhythmias. This effect was more pronounced in the group receiving highest dose equivalent. This suggests that multiple mechanisms beyond the traditional paradigm of beta 1 adrenergic suppression may affect the efficacy of these two widely used beta blockers. (a) Metoprolol succinate selectively blocks beta_1_‐receptors, whereas carvedilol acts on three adrenergic receptors (alpha _1_, beta _1_, and beta _2_) resulting in a greater reduction in the harmful effects of catecholamines on the myocardium.^9^ Cardiac beta receptor density is increased with metoprolol use while it is unaffected by carvedilol.^9^ (b) Carvedilol but not metoprolol blocks the alpha_1_ receptor and lowers plasma endothelin‐1 level; this in turn results in reduced electromechanical stress in the myocardium and thus reduce the risk of arrhythmias(especially in nonischemic CM).^10^, ^11^ (c) Stimulation of beta_2_ receptors by circulating catecholamines can transiently lower the plasma potassium level by enhancing potassium entry into cells, this effect may be arrhythmogenic.^12^, ^13^ Carvedilol, by virtue of its beta_2_ blocking property can mitigate this effect.

The anti‐arrhythmic superiority of carvedilol to metoprolol has been demonstrated in some clinical situations. In a meta‐analysis, carvedilol was superior to metoprolol (tartrate) in reducing the incidence of postoperative atrial fibrillation after cardiac surgery^14^; however, little data exists to support the use of one medication over the other in prevention of ventricular arrhythmias in patients with reduced LV ejection fraction. The efficacy of carvedilol in reducing appropriate ICD therapies was previously demonstrated in a post hoc analysis of the Multicenter Automatic Defibrillator Implantation Trial with Cardiac Resynchronization Therapy.^15^ In this post hoc analysis, carvedilol was associated with a reduction in incidence of ventricular arrhythmias which barely reached statistical significance (0.80 [95% CI: 0.63 to 1.00], *P* = 0.05) compared with metoprolol. However, 12% in the metoprolol arm used the tartrate preparation which is known to be inferior to carvedilol.^16^ In addition, there were significant baseline differences (lesser number of ischemic CM in carvedilol group) which may have affected the outcome. In the current study, there was no significant difference in baseline parameters between carvedilol and metoprolol succinate and patients on metoprolol tartrate were excluded.

In the present study, carvedilol was not associated with a reduction in inappropriate ICD therapy for supraventricular tachyarrhythmias compared to metoprolol succinate. This can be explained by the fact that in clinically used doses, the effects of carvedilol and metoprolol on the atrioventricular node are similar which may result in reduced ventricular rate response in patients with supraventricular arrhythmias.^17^, ^18^, ^19^ The results of this study differ from a prior post hoc analysis of the MADIT CRT study which showed that carvedilol was superior to metoprolol in reducing inappropriate ICD therapies.^20^ The present study included patients with both CRT and non‐CRT devices and may have been underpowered to detect a significant difference.

## 5 STUDY LIMITATIONS

This was a retrospective, nonrandomized, single center study. We studied beta‐blocker type and dose at device implantation (a prespecified intention to treat type of analysis) and about 10% of the cohort switched between the two beta‐blockers during the course of follow‐up. Compliance with beta‐blockers could not be assessed due to the retrospective study design. Programming of ICD therapies was at the discretion of the treating electrophysiologist and may have affected the incidence of the type of therapy administered for ventricular arrhythmias. However, this did not affect the combined endpoint of ICD shocks and ATP. Though the number of patients included was relatively small, this is the first real world, well matched analysis of the efficacy of these drugs in preventing ICD therapies. We limited our analysis to time to event rather than cumulative event rates since patients who receive ICD therapies are often started on other anti‐arrhythmic drugs which preclude assessment of the efficacy of the beta‐blocker alone. We did not collect data on heart rate which is often used as a surrogate marker for efficacy of beta blockade. However, the superiority of carvedilol over metoprolol at the highest dose equivalents suggests that factors other than rate reduction by beta1 antagonism may be at play. Finally, residual confounders not included in the analyses may have biased our results.

## 6 CONCLUSION

Carvedilol improves survival free of appropriate ICD therapy compared with metoprolol succinate in patients with HFrEF. Because both these drugs are now available generically, a pragmatic randomized controlled trial to study the efficacy of these drugs in HFrEF (focused on both arrhythmic and nonarrhythmic outcomes) is warranted.

## CONFLICT OF INTEREST

The authors declare no potential conflict of interests.
